# The Week's Work

**Published:** 1910-07-16

**Authors:** 


### The Week's Work.

### THE BRITISH HOSPITALS ASSOCIATION.

The Honorary Secretaries, Dr. D. J. Mackintosh and Mr. A. W. West, are completing arrangements for a Hospital Conference of chairmen, hospital governors, superintendents, and secretaries in the United Kingdom, to be held at Glasgow at the end of September or beginning of October. The Court of the University of Glasgow at its meeting last week granted the use of the University buildings to the Hospital Conference, and much interest is being taken and hospitality offered with the object of making this meeting successful and enjoyable. The papers already promised include: "The Poor-Law Commission's Report as it affects Voluntary Hospitals," by Dr. C. S. Loch; "The Coming of a Unified County Medical Service and how it will affect the Voluntary Hospital," by Mrs. Sydney Webb; and one from Dr. Nathan Raw, embracing the whole administrative control of poor persons afflicted with tubercle. Two mornings will be devoted to the papers and discussions, whilst the afternoons and evenings will be taken up by visits to the hospitals and entertainments. Sir Henry Burdett has been asked to read a paper upon "The Ideal Arrangement of the Hospital Service for a large Urban Community." Offers of papers from gentlemen connected with hospitals will be gladly received, and should be addressed to the Hon. Secretaries of the British Hospitals Association, 29 Southampton Street, Strand, London, W.C.

### HOSPITAL RATING.

We have always held the view that hospital authorities would be well advised to appeal against the full assessment of hospital buildings whenever such assessments are made by the local authorities. In fact, it seems to us clear that the decision of the Greenwich Rating Authority, with regard to the Dreadnought Hospital many years ago, is the right one for all assessment committees to take when deciding a basis for rating voluntary hospitals. This decision was that the rating authorities should confine the assessment to those portions of the hospital which are beneficially occupied—*i.e.* to the houses or quarters of the resident officers, the medical school buildings, and the wards for paying patients, if such there be. If this practice were to be universally followed, voluntary hospitals would have no cause to complain of the excessive levies which many of them at present have to face. At the West Riding Quarter Sessions recently the trustees of the Bath Hospital and Rawson Convalescent Home, Harrogate, successfully appealed against the poor rates levy. The local authority had fixed the assessment at £727, which the trustees appealed against on the ground that it was far too high, and that any such assessment should be merely nominal, having regard to the work which these institutions do for the community in the district in which they are placed. The institution in question was formerly rated at £50 gross; this was subsequently increased to £500, but on appeal was reduced by the assessment committee to £50, and was then again raised to £727. Last week evidence was given that the institution was not capable of making a profit and that the assessment ought to be nominal. It appeared that the assessment had been raised in consequence of a circular from the Local Government Board calling attention to these small assessments. In the course of the proceedings it was ruled that the case should proceed in order to decide the broad question whether the assessment should be a nominal one or not. It appeared that the assessment of £727 had been arrived at by taking the original cost of the buildings at £36,356, from which cost the surveyor had estimated the present rateable value at £28,350; the site of 4¾ acres was valued at £1,694 per acre, with an annual value of £160, plus 2 per cent. calculated on the value of the buildings, or £567. This gave a total gross annual value of £727 and a net value of £582. The surveyor for the assessment authorities when cross-examined as to the beneficial occupation admitted no profit was made and did not prove any beneficial occupation. After hearing evidence and counsel, the magistrates decided unanimously to reduce the rates to the nominal sum of £50 gross and £40 net. More may be heard of the matter, as the assessment committee propose to take the case to the High Court. Proceedings, as far as they have gone, show that the opinion of fair-minded men of business like the magistrates of the West Riding is opposed to the assessment of voluntary hospitals for any but a nominal amount.

### HOPE HOSPITAL.

To our great regret we have to record the breakdown in health of Dr. Hindshaw, who was recently appointed medical superintendent to the Salford Hope Hospital. Dr. Hindshaw was settling down to his work with commendable success, and we share the great regret expressed by the General Purposes Committee, to whom his resignation was reported on the 8th instant. The task which lay before Dr. Hindshaw in assuming the duties of medical superintendent was a very responsible one, and the fact that he has won the confidence and appreciation of the Guardians and the resident staff in such a short time is the best evidence of his capacity to discharge successfully the arduous duties of this important post. All wish that Dr. Hindshaw's estimate of the serious character of his illness may be negatived by experience, and that we shall have the pleasure of welcoming him back to health at the end of the period of treatment which he has now, unfortunately, to face. We are confident that the sympathy of all hospital workers will be extended to Dr. Hindshaw in the painful circumstances. We hope that some active and experienced member of the medical profession may be found to fill the vacancy at the Hope Hospital, and to continue the good work which Dr. Hindshaw has so successfully initiated.

### CORONERS, JURIES, AND HOSPITALS.

The experience of many hospital workers leads them to regard the work of the coroner's court as not altogether satisfactory, if it is not sometimes even unjust. Much, of course, depends upon the character of the coroner, and, where hospitals are concerned, more upon the profession to which he may belong. In a recent case at Manchester the city coroner has given dissatisfaction to the jury, who had before them the case of a patient treated at the Manchester Royal Infirmary. The jury complain in a letter sent to the Press that they should have been directed at the outset to be generously disposed in their verdict towards the medical staff of the infirmary. They further complain that they were refused an opportunity (1) to hear the evidence of Dr. Jones, who apparently had seen the patient previous to his death, and (2) to question any medical man who attended the case when at the infirmary. They further complain that a rider to their verdict has not been recorded, which declared that the jury were not satisfied that the treatment received was altogether right; that the patient was dismissed from the infirmary at too early an hour in the morning; that he was not sent home in an ambulance; and that death was in some measure due, in their view, to inadequate attendance, which may have been inadvertent, on the part of some person or persons. We await with interest the answer of the coroner, who does not appear, so far as we have been able to ascertain, to have yet replied to the jury's letter to the Press.

### THE RIGHT TO ABUSE THE HOSPITALS.

Recently we called attention to the wisdom of King Edward's Hospital Fund "in insisting that every hospital shall employ at least one almoner who devotes his or her whole time to the work." This week has provided us with a pungent illustration of the muddle which exists where this is not done. The Wigan Infirmary, like the majority of provincial hospitals (which lie, of course, beyond the influence of the King's Fund), complains of congestion in the out-patient department. The hon. medical officer, Dr. Rees, who attends there, has found that he cannot give proper attention to the host of applicants awaiting him. He has considered, therefore, whether the numbers cannot be cut down, or at least sifted; but is pulled up sharply on learning from the Board of Management that while, of course, every applicant bears a "recommend," the responsibility for each "recommend" is held to rest with the giver of it. This simply means that everyone who subscribes enough money to have "recommends" allotted to him buys the right to abuse the hospital. Abuse will not always result, but the absence of any criticism on the part of the hospital over the type of person recommended means that the purchaser of "recommends" can bestow them on whom he will. The situation has to be stated only in plain terms for its absurdity to be obvious. The remedy, of course, is to appoint a hospital almoner who shall supply tactfully that discrimination which is often found wanting in subscribers. If Boards of Management shirk the responsibility of deciding who is ineligible for treatment, the moment they complain of hospital abuse the intelligent public will retort that they have only themselves to thank for it. To throw all responsibility on the subscriber is to invite him to abuse his trust, and it is clear that he will do so from the same motive which led the hospital to give him a free hand—that is, not from malice prepense, but simple laziness.

### A HOSPITAL PEST.

A case of some interest to hospital officers and governors came before the magistrate at Clerkenwell on June 25. A man who described himself as a cabinet maker was charged with unlawfully and by means of false pretences obtaining medical care and attention from the governors of the Royal Free Hospital with intent to cheat and defraud. It appeared from the evidence of one of the residents that a telephone message was received one evening at the hospital purporting to be from a medical man, asking that a patient might be admitted at once. Later on the prisoner presented himself, claimed to be the patient referred to, and tendered a letter with a signature which was afterwards found not to be contained in the Medical Register. He also finally admitted having been the spokesman on the telephone. Having been admitted to the hospital late at night complaining of headache, dizziness, deafness, and double vision following an accident, it was not until the next day that a complete physical examination was made. It was then discovered that no signs of any lesion existed, and the man was discharged as a malingerer, and was arrested by the police on leaving the hospital. According to the detective who gave evidence, the man has been playing this game upon hospitals for three years. There is no reason to doubt that the man is quite able-bodied, and he is the sort of social pest and parasite that deserves a smart sentence. Unfortunately, however, the public-spirited example set by the authorities of the Royal Free Hospital finds no imitators elsewhere, and the magistrate discharged the prisoner on the ground that there was not sufficient evidence of false pretences. Had the other hospitals prosecuted, he said, the man would have been committed for trial. It seems a curious view of the law that a rogue must be prosecuted by two of his victims before he can be punished for fraud, but it is to be supposed that the magistrate knows what he is talking about. In these circumstances it is much to be regretted that no hospital secretary or board in London can be found willing to co-operate in the suppression of an expensive nuisance.

### THE DRUG MARKET.

Few alterations in prices have taken place during the week, and the market remains very quiet. Citric acid is in good demand, and an advance in price is thought to be probable. As was expected, the price of santonin has again been raised, the increase in price being 9d. per lb. Opium is again lower, and a further reduction in the price of morphine is expected; the price of codeine may also be reduced. Glycerin is tending dearer. Senega is a trifle cheaper.

